# ^18^F-FET PET Uptake Characteristics of Long-Term IDH-Wildtype Diffuse Glioma Survivors

**DOI:** 10.3390/cancers13133163

**Published:** 2021-06-24

**Authors:** Lena M. Mittlmeier, Bogdana Suchorska, Viktoria Ruf, Adrien Holzgreve, Matthias Brendel, Jochen Herms, Peter Bartenstein, Joerg C. Tonn, Marcus Unterrainer, Nathalie L. Albert

**Affiliations:** 1 Department of Nuclear Medicine, University Hospital, LMU Munich, 81377 Munich, Germany; Lena.Mittlmeier@med.uni-muenchen.de (L.M.M.); Adrien.Holzgreve@med.uni-muenchen.de (A.H.); Matthias.Brendel@med.uni-muenchen.de (M.B.); Peter.Bartenstein@med.uni-muenchen.de (P.B.); Marcus.Unterrainer@med.uni-muenchen.de (M.U.); 2 Department of Neurosurgery, University Hospital, LMU Munich, 81377 Munich, Germany; Bogdana.Suchorska@med.uni-muenchen.de (B.S.); Joerg.Christian.Tonn@med.uni-muenchen.de (J.C.T.); 3 Institute for Neuropathology and Prion Research, LMU Munich, 81377 Munich, Germany; Viktoria.Ruf@med.uni-muenchen.de (V.R.); Jochen.Herms@med.uni-muenchen.de (J.H.); 4 German Cancer Consortium (DKTK), partner site Munich, German Cancer Research Center (DKFZ), 69126 Heidelberg, Germany; 5 Department of Radiology, University Hospital, LMU Munich, 81377 Munich, Germany

**Keywords:** glioma, FET-PET, BTV, IDH-wildtype, long-term-survivors

## Abstract

**Simple Summary:**

IDH-wildtype (IDHwt) gliomas represent a tumor entity with poor overall survival. Only rare cases have an overall survival over several years. Dynamic and static ^18^F-FET PET is recommended as valuable complementary tool for glioma imaging in gliomas. This study shows that, besides molecular genetic prognosticators, long survival (≥36 months survival) in IDHwt gliomas is associated with a longer time-to-peak and smaller volume on ^18^F-FET PET at initial diagnosis compared to glioma patients with a short-term survival (≤15 months survival). ^18^F-FET uptake intensity and MRI-derived tumor size do not differ in patients with long-term survival compared to patient with a short-term survival.

**Abstract:**

Background: IDHwt diffuse gliomas represent the tumor entity with one of the worst clinical outcomes. Only rare cases present with a long-term survival of several years. Here we aimed at comparing the uptake characteristics on dynamic ^18^F-FET PET, clinical and molecular genetic parameters of long-term survivors (LTS) versus short-term survivors (STS): Methods: Patients with de-novo IDHwt glioma (WHO grade III/IV) and ^18^F-FET PET prior to any therapy were stratified into LTS (≥36 months survival) and STS (≤15 months survival). Static and dynamic ^18^F-FET PET parameters (mean/maximal tumor-to-background ratio (TBR_mean/max_), biological tumor volume (BTV), minimal time-to-peak (TTP_min_)), diameter and volume of contrast-enhancement on MRI, clinical parameters (age, sex, Karnofksy-performance-score), mode of surgery; initial treatment and molecular genetics were assessed and compared between LTS and STS. Results: Overall, 75 IDHwt glioma patients were included (26 LTS, 49 STS). LTS were significantly younger (*p* < 0.001), had a higher rate of WHO grade III glioma (*p* = 0.032), of O(6)-Methylguanine-DNA methyltransferase (*MGMT*) promoter methylation (*p* < 0.001) and missing Telomerase reverse transcriptase promoter (TERTp) mutations (*p* = 0.004) compared to STS. On imaging, LTS showed a smaller median BTV (*p* = 0.017) and a significantly longer TTP_min_ (*p* = 0.008) on ^18^F-FET PET than STS, while uptake intensity (TBR_mean/max_) did not differ. In contrast to the tumor-volume on PET, MRI-derived parameters such as tumor size as well as all other above-mentioned parameters did not differ between LTS and STS (*p* > 0.05 each). Conclusion: Besides molecular genetic prognosticators, a long survival time in IDHwt glioma patients is associated with a longer TTP_min_ as well as a smaller BTV on ^18^F-FET PET at initial diagnosis. ^18^F-FET uptake intensity as well as the MRI-derived tumor size (volume and maximal diameter) do not differ in patients with long-term survival.

## 1. Introduction

IDH-wildtype (IDHwt) diffuse gliomas represent the tumor entity with one of the worst clinical outcomes. Only rare cases comprise an extensive survival over several years [1]. However, it still remains unclear which clinical and molecular genetic features are associated with the occurrence of such an extensively long survival [2]. Recent studies aimed to identify biological and molecular genetic differences between long-term survivors (LTS) and short-term survivors STS, but could not find any significant clusters of genomic events between STS and LTS [2].

In clinical routine, molecular imaging using positron-emission-tomography (PET) with radiolabeled amino acids such as *O*-(2-^18^F-fluoroethyl)-L-tyrosine (^18^F-FET) has gained increasing importance for the noninvasive evaluation and characterization of primary brain neoplasms on a molecular level beyond magnetic-resonance-imaging (MRI); MRI is the standard for brain tumor imaging due to its soft-tissue contrast, spatial resolution, and widespread availability. Nonethteless, MRI imaging has major drawbacks such as a rather low sensitivity and specificity for neoplastic tissue that hampers the differentiation of vital tumor and nonneoplastic lesion, the identification of tumor extent (particularly in nonenhancing tumors) and, especially, the differentiation of tumor progression from treatment-related changes [3]. Hence, PET imaging has been recommended by the RANO working group as valuable complementary tool for glioma imaging [4], e.g., for planning of surgery, therapy monitoring or prognostication [5,6,7,8,9,10].

In the light of the rare occurrence of LTS in IDHwt glioma, we aimed to identify their typical uptake characteristics on dynamic ^18^F-FET PET at initial diagnosis and compared them to short-term survivors.

## 2. Materials and Methods

### 2.1. Patients

Patients with histologically confirmed newly diagnosed glioma (WHO grade III/IV), available molecular genetic profile, ^18^F-FET PET scan prior to stereotactic biopsy or surgical resection were retrospectively identified. Based on the common consent [11,12,13], patients with a survival time ≥36 months were defined and included as LTS. In order to extract typical imaging characteristics associated with the phenomenon of long-term survival, we compared LTS to a matched group of patients with diametral extreme survival, i.e., STS. Patients were included as STS, if they had a confirmed survival of ≤15 months, which is a cut-off based on historic data [14]. Firstly, characteristics of LTS and STS were compared directly. In a second step, neuropathologically matched groups from the LTS and STS subgroups were built in order to take into account molecular genetic and histologic parameters.

Overall, in the institutional data base, 182 cases with de-novo IDH-wt glioma and ^18^F-FET PET prior to any therapy were identified. Of those, 107 patients presented with a survival of 15–36 months or were lost to follow-up without documented death < 36 months or showed incomplete molecular genetic/clinical features. Overall, 49 patients were included in the STS cohort and 26 patients were included in the long-term survivor group. All patients gave written informed consent prior to the PET examination as part of the clinical routine. Ethical approval for retrospective data analysis was given by the institutional review board of the LMU.

### 2.2. ^18^F-FET PET Image Acquisition and Data Analysis

^18^F-FET PET scans were performed at the Department of Nuclear Medicine, LMU. After a 15-min transmission scan with a ^68^Ge rotating rod source, approximately 180 MBq of ^18^F-FET were injected. Data of the forty-minutes dynamic ^18^F-FET PET scans were acquired using an ECAT Exact HR+ scanner (Siemens Healthineers, Erlangen, Germany). After tracer injection up to 40 min post injection dynamic emission recording was accomplished in 3-D mode consisting of 16 frames (7 × 10 s; 3 × 30 s; 1 × 2 min; 3 × 5 min; 2 × 10 min). Using a 5 mm Hann Filter two-dimensional filtered back-projection was used for image reconstruction, corrected for photon attenuation and model-based scatter. For further evaluation, images were transferred to a Hermes workstation (Hermes Medical Solutions, Stockholm, Sweden).

The mean background activity (BG) was assessed using 6 large crescent-shaped regions of interests (ROI) in the frontal lobe of the healthy contralateral hemisphere as previously published [15]. BTV was estimated by a semiautomatic threshold-based delineation of a volume of interest (VOI) using a standardized uptake value (SUV) threshold of 1.6 x BG as described as optimal threshold [16]. 

### 2.3. Histological Confirmation, Tumor Grading and Molecular Genetic Analysis

Stereotactic biopsy procedures and microsurgical resections were performed at the Department of Neurosurgery, LMU Munich, Germany. Histopathological as well as molecular genetic evaluations were performed at the Institute of Neuropathology, LMU Munich, Germany, according to the updated 2016 WHO classification [17]. For further specification regarding the histopathological workup, see also [18].

### 2.4. MRI

Patients underwent MRI (1.5 T or 3 T) with a head coil before and after administration of a gadolinium-based contrast agent (T1- and T2-weighted). Axial T1-weighted images were obtained from the second cervical vertebral body to the vertex. The maximum diameter of the entire lesion including all contrast-enhancing areas was assessed. In order to exclude cystic or necrotic tumor parts from the measurements, the volume of the contrast enhancement was evaluated by slice-by-slice volumetric procedure excluding necrotic parts. Additionally, the tumor localization on MRI was divided into “deep seated” and “lobar” localization.

### 2.5. Statistics

SPSS for Windows (version 25.0; SPSS, Chicago, IL, USA) was used for statistical analyses. Normal distribution was assessed using the Shapiro-Wilk-test. The Chi-square test was used to assess the distribution of non-continuous parameter between two groups. The unpaired and paired Mann-Whitney-U test was used to compare independent and not-normally distributed continuous parameters. Statistical significance was defined as a two-tailed *p*-value < 0.05.

## 3. Results

### 3.1. Patients

Overall, 75 patients (30 female, 45 male; median age 61.9 years (33.5–77.2); median Karnofsky performance score (KPS) 80.0 (40.0–100.0)) with newly diagnosed IDHwt glioma were included. Of these, 49 (65.3%) patients underwent stereotactic biopsy and 26 (34.7%) a microsurgical resection at initial diagnosis. Histological workup revealed 20/75 (37.5%) WHO grade III anaplastic astrocytomas and 55/75 (62.5%) WHO grade IV glioblastomas. *MGMT* promoter methylation was present in 38/75 (50.7%) patients, 37/75 (49.3%) patients presented with an unmethylated *MGMT* promotor. TERTp mutations were found in 60/75 (81.1%) patients, while 14/75 (18.9%) patients showed no TERTp mutation (see also Table 1). Among the 75 included patients, 49/75 (65.3%) patients were defined as STS, 26/75 (34.7%) patients were defined as LTS. Initial therapies following resection/biopsy consisted of chemotherapy (6/75, 8.0%), radio-/chemotherapy (51/75, 68.0%), radiotherapy (14/75, 18.7%) and brachytherapy (1/75, 1.3%). Three patients died before initiation of a tumor-specific therapy (3/75, 5.1%). For further specifications see also Table 1.

### 3.2. Imaging Characteristics

#### 3.2.1. ^18^F-FET PET

Overall, 74/75 (98.7%) patients were classified as ^18^F-FET-positive, while 1 patient was classified as ^18^F-FET-negative and showed even a photopenic defect with a ^18^F-FET uptake below background activity. Overall, the median TBR_max_ was 3.1 (1.5–6.1) and the median TBR_mean_ was 2.0 (0.8–2.9). The median BTV was 22.8 mL (0.0–133.3 mL) and the median TTP_min_ in the dynamic analysis was 12.5 (7.5–35.0) min; for further specifications see also Table 1.

#### 3.2.2. MRI

Overall, 66/75 patients (88.0%) showed contrast enhancement on MRI; among these, the median diameter of CE was 2.5 cm (0.0–7.2 cm) and the median volume of CE was 5.7 mL (0.0–128.7 mL; see Table 1). 54/75 (72.0%) patients showed a lobar tumor localization, 21/75 (28.0%) had a deep-seated glioma. 9 patients showed a non-contrast-enhancing glioma on MRI; of these, 2/9 patients (22.2%) were STS and 7/9 patients (77.8%) were LTS. 8/9 (88.8%) patients with non-contrast-enhancing glioma were ^18^F-FET-positive comprising a BTV range of 0.0–27.7 mL.

### 3.3. Comparison of LTS and STS—Clinical Features

Comparing LTS and STS, LTS had a significantly lower proportion of WHO grade IV as opposed to grade III tumors (15/26 (57.7%) vs. 40/49 (81.6%); *p* = 0.032) and were significantly younger (median age 55.8 (33.5–71.9) vs. 64.0 (40.5–77.2) years, *p* < 0.001). In LTS, there was a significantly higher proportion of patients with *MGMT*-methylation (21/26 (80.8%) vs. 17/49 (34.7%), *p* < 0.001) and a significantly lower proportion of TERTp-mutations (16/26 (61.5%) vs. 44/49 (89.8%), *p* = 0.004). Moreover, LTS had a significantly higher proportion of patients undergoing primary surgery than STS (14/26 (53.8%) vs. 12/49 (24.5%), *p* = 0.021) (see Table 1). Concerning initial therapies, there was a higher proportion of patients undergoing combined radiochemotherapy in LTS (23/26 (88.6%) vs. 28/49 (57.1%), *p* = 0.040), see also Table 2.

### 3.4. Comparison of LTS and STS—Imaging Features

**Figure 1:**
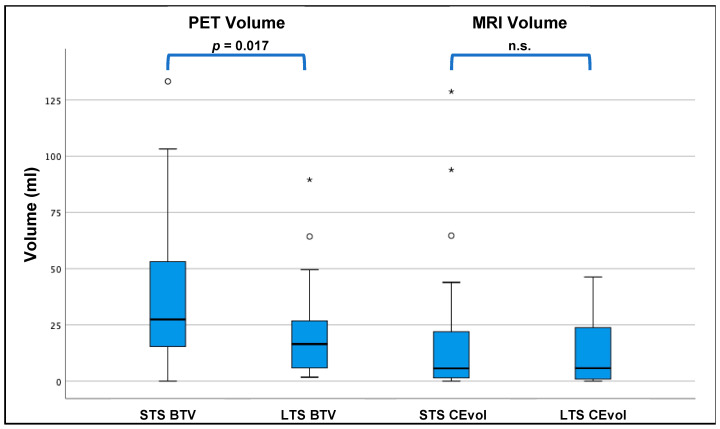
Comparison of PET-based and MRI-based tumour volumes in LTS vs. STS: the PET-based BTV was significantly smaller in the LTS group compared to the STS group (16.5 vs. 27.4 mL; *p* = 0.017), whereas the volume of CE (CEvol) on MRI was comparable between STS and LTS (5.7 vs. 5.8 mL, *p* = 0.802). */° = outliers. N.s. = not significant.

**Figure 2:**
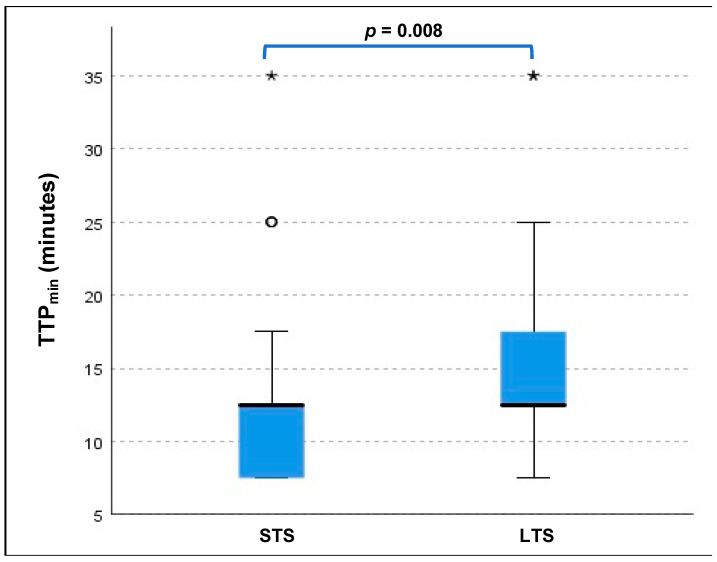
LTS displayed a significantly longer TTP_min_ (median 12.5 min (7.5–35.0); mean 13.3 ± 5.1 min) than STS patients (median 12.5 min (7.5–35.0); mean 9.9 ± 6.3 min; *p* = 0.008. */° = outliers.

LTS had a significantly smaller median BTV on PET compared to STS (16.5 mL (1.75–89.5 mL) vs. 27.4 mL (0.0–133.3 mL), *p* = 0.017, see also Figure 1 and Table 1) as well as a significantly longer TTP_min_ (mean 13.3 ± 5.1 min vs. mean 9.9 ± 6.3 min, *p* = 0.008, see also Figure 2). The other PET-derived parameters of uptake intensity (TBR_mean_, TBR_max_) as well as all MRI parameters showed no significant differences between LTS and STS (*p* > 0.05 each), see also Table 1.

BTV was significantly larger than the volume of CE in the LTS (median 16.5 mL (1.8 89.5 mL) vs. 5.8 mL (0.0–46.3 mL); *p* = 0.006) as well as in the STS group (median 27.4 mL (0.0–133.2 mL) vs. 5.7 mL (0.0–128.7 mL); *p* < 0.001 (Figure 2).

With regard to the localization of the tumor (lobar vs. deep-seated), no differences were found between LTS and STS (*p* > 0.05 each): 20/26 (76.9%) patients with LTS had a lobar localization, while 6/26 (23.1%) patients had a deep-seated location. 34/49 (69.4%) STS patients had a lobar location of the tumor while 14/49 (30.6%) STS patients had a deep-seated tumor.

### 3.5. Differences between LTS and STS Matched for Molecular Genetic and Histologic Parameters

When comparing LTS and STS consisting of groups with a directly matched distribution of histological and molecular genetic factors (WHO grade: *p* = 0.751, *MGMT*-status: *p* = 0.111, TERTp-status: *p* = 0.159), comparable results could be obtained as reported above: LTS still were significantly younger than STS (median 57.3 years (33.5–66.0) vs. 67.0 years (47.1–74.2), *p* = 0.002) and had a higher proportion of patients undergoing surgical resection (11/21 (52.4%) vs. 3/21 (14.2%) in the STS group; *p* = 0.020). Concerning further adjuvant therapies, there was no significant difference between LTS and STS. On imaging, LTS showed again a significantly smaller tumor volume on PET (median BTV 16.1 mL (1.8–49.5 mL) vs. 25.8 mL (0.0–133.3 mL), *p* = 0.028). Also, TTP_min_ was significantly longer in the LTS group (12.5 min (7.5–35.0, mean 14.0 ± 6.8) vs. 12.5 min (7.5–17.5, mean 9.3 ± 3.7), whereas the other PET-derived parameters (TBR_mean_, TBR_max_) still showed no significant differences (*p* > 0.05 each). Also, all MRI derived parameters were indifferent between these matched groups (CE, diameter, volume) with a *p* > 0.05 each. For further specifications, see Table 3 and Table 4.

### 3.6. Analysis of Inter-Correlation

All results of the inter-correlation analysis are displayed in Table 5. BTV was only weekly correlated with the patients’ age (*r* = 0.358, *p* = 0.002) and KPS (*r* = −0.239, *p* = 0.039). Also, there was only a weak to moderate correlation to the MRI-derived metric parameters such as the volume of CE (*r* = 0.580, *p* < 0.001) and other quantitative PET parameters such as TBR_mean_ (*r* = 0.366, *p* < 0.001). WHO grade III gliomas showed a significantly lower BTV compared to WHO grade IV gliomas (median 9.1 vs. 26.4 mL, *p* = 0.023). TTP_min_, on the contrary, did not show any inter-correlation with these parameters (*p* > 0.05 each).

## 4. Discussion

IDHwt diffuse gliomas are aggressive, incurable malignancies with poor survival of approximately 15 months only. Just a very small proportion of patients presents with a long-term survival of several years [2,13]. The occurrence of these LTS in IDHwt gliomas is not yet understood. This is the first analysis to evaluate differences of LTS in amino acid PET and MRI in a well-defined cohort consisting of newly diagnosed IDHwt gliomas and ^18^F-FET PET imaging prior to any further therapies.

**Figure 3:**
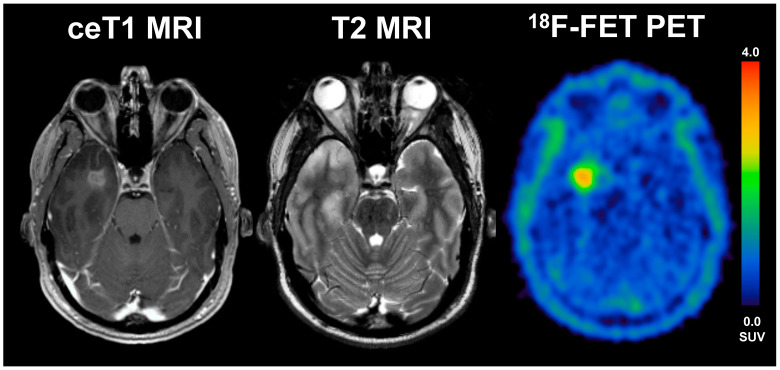
A 33-year old male patient with glioblastoma (WHO grade IV, IDH wildtype, *MGMT* methylated) and an overall survival of 47 months (LTS). The diameter of CE on T1 MRI was 17 mm, volume of CE was 28 mL, the BTV in FET-PET was 16 mL, the TTP_min_ was 17.5 min and TBR_max_ 4.1. The patient underwent radio-chemotherapy.

**Figure 4:**
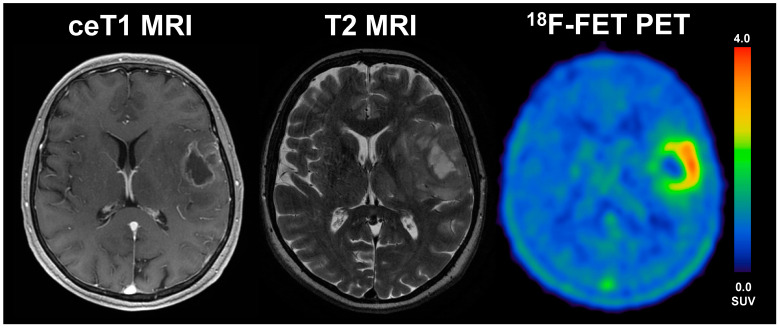
57-year old female with glioblastoma (WHO grade IV, IDH wildtype, *MGMT* unmethylated) and an overall survival of 4.9 months (STS). The diameter of CE on T1 MRI was 31 mm, the volume of CE was 6 mL, the BTV in FET-PET was 39.1 mL, the TTP_min_ was 12.5 min and TBR_max_ was 3.9. The patient underwent radiochemotherapy.

Overall, we found a significantly smaller BTV on PET in the LTS patients compared to STS patients, as the median BTV in the STS group was around 1.7-fold higher than in LTS patients (median 27.4 vs. 16.5 mL, *p* = 0.017). On MRI, the routinely applied clinical gold standard for glioma imaging, however, we could not find any significant differences between LTS and STS patients at all; here, the volume and diameter of CE were comparable between LTS and STS patients (see Figure 2), a finding that is in line with data correlating the volume/diameter of CE with the overall survival in glioblastoma patients [19]; this phenomenon might most likely be linked to a underestimation of the “real tumor extent” as displayed by contrast enhancement compared to PET imaging. In direct comparison to MR imaging, the PET-derived BTV was significantly larger than the MRI-based tumour volume, which was evident in the overall group, but also in both subgroups of STS and LTS patients. However, the difference of BTV and CE was distinctly smaller in LTS patients compared to STS patients, where the median BTV was distinctly larger (STS: median 5.7 vs. 27.4 mL; *p* < 0.001; LTS: median 5.8 vs. 16.5 mL; *p* = 0.006); direct comparisons are displayed in Figure 2. Patient examples can be found in Figure 3 and Figure 4).

In the current literature, BTV was described as independent prognostic factor in newly diagnosed glioblastoma for patient outcome: for example, Poulsen et al. could show that large BTV on ^18^F-FET PET is associated with poor overall survival and short progression free survival in a cohort consisting of 146 glioblastoma patients prior to radiation therapy with concomitant and adjuvant temozolomide [20].

Beyond differences on PET-derived BTV, we observed significant differences with regard to ^18^F-FET uptake dynamics, as LTS patients comprised significantly longer TTP_min_ values compared to STS patients. In the current literature, a short TTP_min_ was shown to be associated with worse outcome in patients with newly diagnosed astrocytic HGG and LGG [7]. Vettermann et al. could show that the IDH-mutational status can be detected by dynamic PET with a high diagnostic accuracy, as a short TTP_min_ is associated with an IDHwt status while, vice versa, a long TTP_min_ is associated with an IDH-mutation [21]. But even within our patient group of IDHwt gliomas only, we could find a significant TTP_min_ difference between LTS and STS with higher values of TTP_min_ in LTS, a finding comparable to the recently published results by Bauer et al. regarding IDHwt gliomas and ^18^F-FET PET imaging [22].

**Figure 5:**
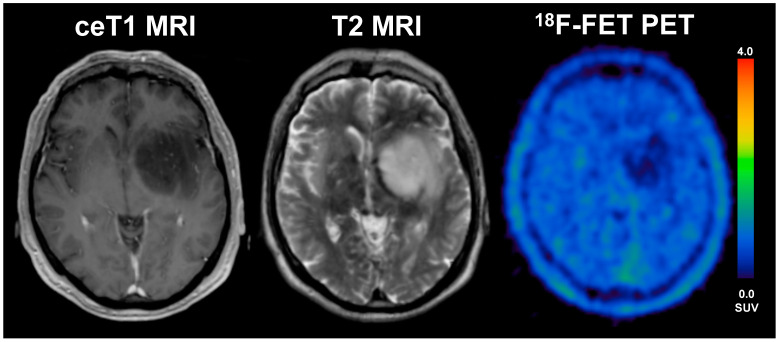
56-year old male patient with anaplastic astrocytoma (WHO grade III, IDH wildtype, *MGMT* unmethylated, TERTp wildtype) and a photopenic aspect on ^18^F-FET PET. Overall survival was 10 months only (STS) under single therapy with radiotherapy.

Interestingly, no differences between LTS and STS were observed with regard to the static uptake intensity parameters on PET. Of note, one STS patient was rated ^18^F-FET-negative with ^18^F-FET-uptake even lower than the respective background activity, i.e., photopenic glioma. In line with the current literature describing that photopenic tumors could have a high risk for harboring aggressive tumors [23,24,25], the current patient with photopenic, IDHwt glioma on ^18^F-FET PET presented also with poor OS, subsequently classified as STS. This finding underlines that photopenic gliomas should be managed more actively (see Figure 5).

Assessing further clinical parameters beyond imaging, LTS patients were significantly younger than STS, a finding that is in line with the current literature [2]. Other characteristics that were previously described to be associated with LTS were female sex and higher Karnofsky performance score [2,26,27]. While we could not find any gender differences in our LTS and STS cohorts, a trend towards a higher initial Karnofsky performance score was indeed seen in our LTS, although marginally not reaching the level of significance (i.e., *p* = 0.056). In our patient cohort, localization of the tumor (lobar vs. deep-seated) was equally distributed between LTS and STS groups, indicating that the localization of the tumor per se may not determine the patients’ survival.

Taking a closer look onto the molecular genetic features, a significantly lower proportion of TERTp mutations was found in our LTS group compared to STS; presence of a TERTp mutation has been associated with poorer OS [2,28] in IDHwt glioma/glioblastoma. Interestingly, *TERTp* mutations seem to be closely associated to the respective WHO grades, i.e., higher tumor grades comprise a higher rate of TERTp mutations than in gliomas with a lower WHO grade [29,30], at least in astrocytic tumors [31], as present in the current cohort. This is in line with a higher proportion of WHO grade IV in STS in this study. However, the prognostic relevance of the WHO grades in IDHwt gliomas remains a topic of debate [32,33,34,35,36]. In line with improved survival in patients with *MGMT*-methylation [2], a higher proportion of methylated *MGMT* promoter was present in LTS patients.

Beyond the scope of imaging and molecular genetics, the subsequent therapies after biopsy or surgery have to be taken into account. In the cohort of LTS, a significantly higher proportion of patients received a multimodal therapy consisting of combined radiochemotherapy, which represents the current standard of care in IDHwt gliomas [37]. Vice versa, a single therapy was performed more often in the STS group, which may be considered—from a current point of view—as a clear “undertreatment”. This could of course, have impacted the short survival of STS patients, however, does still not explain the phenomenon of the long survival of our LTS patients in consideration of currently applied standard treatment regimens [38]. Moreover, as patients with current “undertreatment” were present in the LTS cohort as well. Nonetheless, it needs to be discussed, whether downscaled therapy regimens are a cause or consequence of the particular poor clinical condition with consecutive inferior clinical outcome or even a mutual causation, as some patients are not eligible for standard of care treatments in a real-world clinical scenario, e.g., in elder patients with extensive tumor load and unfavorable molecular genetic profile or patients with unexpectedly rapid tumor progression prior to treatment initiation. Given the retrospective clinical data from a real-world clinical scenario, we cannot fully elucidate the influence of the particular treatment regimens on the phenomenon of LTS patients with IDHwt glioma. This is the case for both scenarios: the occurrence of STS with current standard of care treatment and—vice versa—the occurrence of LTS with clear undertreatment. However, the primary aim of this analysis was not to evaluate the specific clinical benefit of particular therapy regimens, but to assess and describe fixed tumor characteristics that are correlated with the phenomenon of LTS patients; this is especially the case for molecular imaging features derived from ^18^F-FET PET beyond the scope of clinical and molecular genetic factors.

Therefore, to finally account for potential imbalances and inter-correlations caused by diverging molecular genetic profiles between the two groups and the impact of molecular genetics on survival, we performed the same comparisons in a matched control group with comparable distributions of *MGMT*-methylations, TERTp mutations and WHO grades. Interestingly, even after accounting for these molecular genetic parameters lower BTV, longer TTP_min_, open tumor resection and lower median age remained characteristics associated with LTS. Of note, MRI-based features and clinical parameters were still comparably distributed between the two adjusted groups. In direct comparison with the other parameters, a larger BTV in WHO grade IV tumors compared to WHO grade III in the inter-correlation analysis has to be noted; however, BTV still remained significantly smaller in LTS compared to STS after matching for molecular genetics including WHO grades so that only a rather small inter-correlation—if any—could be assumed. Expectedly, BTV showed only week to moderate correlation to other volumetric/morphological parameters such as CE_vol_ and only week to moderate correlation to age and KPS. TTP_min_, however, did not show any inter-correlation to other parameters.

## 5. Conclusions

This is the first analysis to evaluate characteristic features of ^18^F-FET PET in newly diagnosed IDHwt gliomas with a long survival of more than 36 months. LTS are characterized by a significantly smaller initial PET-based tumor volume and a longer TTP_min_ on dynamic ^18^F-FET PET, while MRI-based parameters of the tumour size are not different between LTS and STS. These findings remain significant even after matching for molecular genetics and histology. Overall, imaging parameters derived from dynamic ^18^F-FET PET may provide additional prognostic information beyond molecular biomarkers in newly diagnosed IDHwt glioma.

## Figures and Tables

**Table 1:** Overview and overview differences STS and LTS (15 vs. 36 months).

Parameters	Overall (*n* = 75) [Median (Range)]	STS (*n* = 49) [Median (Range)]	LTS (*n* = 26) [Median (Range)]	Significance (LTS vs. STS)
WHO grade (III/IV)	20 (26.7%)/55 (73.3%)	9 (18.4%)/40 (81.6%)	11 (42.3%)/15 (57.7%)	*p* = 0.032
*MGMT* (methyl./unmethyl.)	38 (50.7%)/37 (49.3%)	17 (34.7%)/32 (65.3%)	21 (80.8%)/5 (19.2%)	*p* < 0.001
TERTp (mutation/wildtype) *	60 (81.1%)/14 (18.9%) *	44 (89.8%)/4 (10.2%)	16 (61.5%)/10 (38.5%)	*p* = 0.004
KPS	80 (40.0–100.0)	80.0 (40.0–100.0)	90.0 (70.0–100.0)	*p* = 0.056
Age [yrs]	61.9 (33.5–77.2)	64.0 (40.5–77.2)	55.8 (33.5–71.9)	*p* = 0.001
Sex (m/f)	45 (60.0%)/30 (40.0%)	31 (63.3%)/18 (36.7%)	14 (53.8%)/12 (46.2%)	*p* = 0.466
CE (y/*n*)	66 (88.0%)/9 (12.0%)	47 (95.9%)/2 (4.1%)	19 (73.1%)/7 (26.9%)	*p* = 0.070
Diameter CE [cm]	25.0 (0.0–72.0)	27.0 (0.0–72.0)	24.5 (0.0–62.0)	*p* = 0.406
Volume CE [ml]	5.7 (0.0–128.7)	5.7 (0.0–128.7)	5.8 (0.0–46.3)	*p* = 0.802
Surgery/Biopsy	26 (34.7%)/49 (65.3%)	12 (24.5%)/37 (75.5%)	14 (53.8%)/12 (46.2%)	*p* = 0.021
^18^F-FET-positive (y/*n*)	74 (98.7%)/1 (1.3%)	48 (98.0%)/1 (2.0%)	26 (100.0%)/0 (0.0%)	*p* = 1.000
TBR_mean_	2.0 (0.8–2.9)	2.0 (0.8–2.9)	1.9 (1.7–2.6)	*p* = 0.227
TBR_max_	3.11 (1.5–6.1)	3.2 (1.5–6.1)	2.9 (1.9–4.9)	*p* = 0.127
BTV [ml]	22.8 (0.0–133.26)	27.4 (0.0–133.2)	16.5 (1.8–89.5)	*p* = 0.017
TTP_min_ [min]	12.5 (7.5–35.0) mean/SD: 13.9 ± 6.4	12.5 (7.5–35.0) mean/SD: 9.9 ± 6.3	12.5 (7.5–35.0) mean/SD: 13.3 ± 5.1	*p* = 0.008

* TERT available in 74/75 patients, the 1 patient without TERT status is in the STS-group.

**Table 2:** Differences between STS and LTS: initial therapy.

Therapy	STS (*n* = 49)	LTS (*n* = 26)
Combined radiochemotherapy	28 (57.1%)	23 (88.6%)
Chemotherapy	5 (10.2%)	1 (3.8%)
Radiotherapy	13 (26.5%)	1 (3.8%)
Brachytherapy	0 (0%)	1 (3.8%)
None	3 (6.2%)	0 (0%)
Significance	*p* = 0.040	

**Table 3:** Overview patients matched for WHO grade and molecular genetics and overview differences STS and LTS (≤15 vs. ≥36 months).

Parameters	Overall (*n* = 42) [Median (Range)]	STS (*n* = 21) [Median (Range)]	LTS (*n* = 21) [Median (Range)]	Significance (LTS vs. STS)
WHO grade (III/IV)	16 (38.1%)/26 (61.9%)	7 (33.3%)/14 (66.7%)	9 (42.9%)/12 (57.1%)	*p* = 0.751
*MGMT* (methyl./unmethyl.)	26 (61.9%)/16 (38.1%)	10 (47.6%)/11 (52.4%)	16 (76.2%)/5 (23.8%)	*p* = 0.111
TERTp (mutation/wildtype)	31 (73.8%)/11 (26.2%)	18 (85.7%)/3 (14.3%)	13 (61.9%)/8 (38.1%)	*p* = 0.159
KPS	90.0 (70.0–100.0)	80.0 (70.0–100.0)	90.0 (70.0–100.0)	*p* = 0.172
Age [yrs]	60.0 (33.5–74.2)	67.0 (47.1–74.2)	57.3 (33.5–69.0)	*p* = 0.002
Sex (m/f)	27 (64.3%)/15 (35.7%)	14 (66.7%)/7 (33.3%)	13 (61.9%)/8 (38.1%)	*p* = 1.000
CE (y/*n*)	35 (83.3%)/7(16.7%)	19 (90.5%)/2 (9.5%)	16 (76.2%)/5 (23.8%)	*p* = 0.410
Diameter CE [cm]	24.5 (0.0–72.0)	27.0 (0.0–72.0)	24.0 (0.0–61.0)	*p* = 0.554
Volume CE [ml]	5.5 (0.0–128.7)	4.4 (0.0–128.7)	5.7 (0.0–46.3)	*p* = 0.830
Surgery/Biopsy	14 (33.3%)/28 (66.7%)	3 (14.2%)/18 (85.7%)	11 (52.4%)/10 (47.6%)	*p* = 0.020
^18^F-FET-positive (y/*n*)	41 (97.6%)/1 (1.6%)	20 (95.2%)/1 (4.8%)	21 (100%)/0 (0%)	*p* = 1.000
TBR_mean_	2.0 (0.8–2.9)	2.1 (0.8–2.9)	1.9 (1.7–2.6)	*p* = 0.414
TBR_max_	3.0 (1.5–6.1)	3.1 (1.5–6.1)	2.8 (1.9–4.9)	*p* = 0.333
BTV [ml]	19.4 (0.0–133.3)	25.8 (0.0–133.2)	16.1 (1.8–49.5)	*p* = 0.028
TTP_min_ [min]	12.5 (7.5–35.0) mean/SD: 14.5 ± 6.6	12.5 (7.5–17.5) mean/SD: 9.3 ± 3.7	12.5 (7.5–35.0) mean/SD: 14.0 ± 6.8	*p* = 0.013

**Table 4:** Differences between STS and LTS (matched for WHO grade and molecular genetics): initial therapy.

Therapy	STS (*n* = 21)	LTS (*n* = 21)
Combined radiochemotherapy	11 (52.4%)	19 (95.2%)
Chemotherapy	3 (14.3%)	1 (4.8%)
Radiotherapy	6 (28.5%)	1 (0%)
None	1 (4.8%)	0 (0%)
Significance	*p* = 0.090	

**Table 5:** Direct comparison/correlation of BTV/TTP min with other parameters.

Heading	BTV [ml/r-Value]	Significance	TTP_min_ [min/r-Value]	Significance
**Age**	r = 0.358	*p* = 0.002 *	r = −0.075	*p* = 0.528 *
**Sex** (m/f)	19.7 vs. 26.8 ml	*p* = 0.423 °	12.5 vs. 12.5 min	*p* = 0.913 °
**WHO grade** (III/IV)	9.1 vs. 26.4 ml	*p* = 0.023 °	12.5 vs. 12.5 min	*p* = 0.110 °
**Localization** (lobar/deep seated)	21.1 vs. 25.8 ml	*p* = 0.558 °	12.5 vs. 12.5 min	*p* = 0.704 °
**Mode of therapy** (none/RCT/RT/BT)	23.4 vs. 26.4 vs. 22.7 vs. 9.8 ml	*p* = 0.507 ^	12.5 vs. 12.5 vs. 7.5 vs. 12.5 min	*p* = 0.928 ^
**Biopsy/Surgery**	19.0 vs. 26.6 ml	*p* = 0.744 °	12.5 vs. 12.5 min	*p* = 0.512 °
**KPS**	r = −0.239	*p* = 0.039 *	r = −0.016	*p* = 0.891 *
**MGMT** (unmethylated/methylated)	25.8 vs. 22.6 ml	*p* = 0.909 °	12.5 vs. 12.5 min	*p* = 0.353 °
**TERT** (mutant/wildtype)	24.0 vs. 19.9 ml	*p* = 0.529 °	12.5 vs. 12.5 min	*p* = 0.157 °
**CE_diameter_**	r = 0.674	*p* < 0.001 *	r = −0.124	*p* = 0.293 *
**CE_volume_**	r = 0.580	*p* = 0.000 *	r = −0.098	*p* = 0.408 *
**TBR_mean_**	r = 0.366	*p* < 0.001 *	r = −0.185	*p* = 0.114 *
**TBR_max_**	r = 0.439	*p* < 0.001 *	r = −0.170	*p* = 0.148 *

Displayed as median/r-value; * Pearson-correlation coefficient; ° Wilcoxon test; ^ Kruskal-Wallis test; RCT = combined radiochemotherapy; RT = radiotherapy; BT = brachytherapy.

## Data Availability

The data presented in this study are available on reasonable request from the corresponding author.
